# Plant-Based Films and Hydrogels for Wound Healing

**DOI:** 10.3390/microorganisms12030438

**Published:** 2024-02-21

**Authors:** Ana I. Lopes, Maria M. Pintado, Freni K. Tavaria

**Affiliations:** Centro de Biotecnologia e Química Fina—Laboratório Associado, Escola Superior de Biotecnologia, Universidade Católica Portuguesa/Porto, Rua Diogo Botelho, 1327, 4169-005 Porto, Portugal; anlopes@ucp.pt (A.I.L.); mpintado@ucp.pt (M.M.P.)

**Keywords:** wounds, wound healing, films, hydrogels, essential oils, plant extracts, skin microbiota

## Abstract

Skin is constantly exposed to injury and infectious agents that can compromise its structural integrity and cause wounds. When this occurs, microorganisms from the skin microbiota and external bacteria and fungi can penetrate the wound and cause an infection, which complicates the healing process. Nowadays, there are several types of wound dressings available to treat wounds, some of which are incorporated with antimicrobial agents. However, the number of microorganisms resistant to these substances is rising. Therefore, the search for new, natural alternatives such as essential oils (EOs) and plant extracts (PEs) is on the rise. However, these substances present some limitations (poor bioavailability and poor target capacity), which limits their efficiency. Their incorporation in formulations in the form of films and hydrogels (HGs) can help to overcome these issues and may be a potential alternative to the current treatments. HGs and films incorporated with PEs and EOs have antimicrobial activity, promote the viability of skin cells and fibroblast migration, and are non-toxic and biocompatible. This review discusses the use of films and HGs for the topical delivery of EOs and PEs for wound treatment and their formulations as effective wound dressings, while debating some mechanisms and biological properties to elucidate their presumptive clinical relevance and possible applications.

## 1. Introduction

Skin, the largest and outermost organ of the human body, acts as a barrier protecting the muscles, bones, ligaments, and internal organs from biological, chemical, mechanical, and physical threats [1,2]. The constant exposure of the skin to injury and infectious agents can result in the disruption of its normal anatomical structure, causing wounds [3].

Wounds are breaks or defects in the skin caused by thermal or physicochemical damage. They can be classified as acute or chronic, depending on the repair process [4,5]. Acute wounds are injured tissues that usually achieve complete healing within a period of 8 to 12 weeks. In contrast, chronic wounds appear because of diseases such as cancer, diabetes, venous or arterial vascular insufficiency, and pressure necrosis. They need an extended healing time (beyond 12 weeks), often failing to reach a normal healthy state [4,5]. Wounds are also classified based on the affected skin layers and areas. Thus, superficial wounds are those that only involve the skin surface; partial thickness wounds are injuries that affect the epidermis, deeper dermal layers, blood vessels, sweat glands, and hair follicles; and full-thickness wounds are the ones where subcutaneous fat or deeper tissue, epidermis, and dermis are injured [4]. Chronic wounds, such as venous ulcers, pressure sores, and diabetic foot ulcers, represent a major health problem affecting millions of people worldwide and result in billions of dollars of costs for the national health services [6]. 

Burns are serious injuries (wounds) that can cause extreme pain and possibly death. These skin lesions are among the most complex to clinically evaluate and manage. In fact, in addition to pain, they present challenges in restoring patient functionality and cosmetic repair [7,8]. Acute burns lead to a sudden influx of inflammatory cytokines and growth factors. Burns that affect large areas usually result in several complications, such as hypertrophic scarring, facial disfigurement, and loss of muscle and function. They can also be responsible for invisible psychological sequelae [7,8]. A serious complication of acute wounds and burns is sepsis and septic shock. These two phenomena account for approximately 30 million cases per year worldwide, with approximately 6 million being fatal [9].

This review will focus on the use of EOs and PEs in the form of films and hydrogels for the treatment and management of wounds. Due to the lack of recent review papers in this area, it seems important to analyze the studies on this area and identify the research gaps. So, this work will also provide a state-of-the-art review on natural and biodegradable formulations for the delivery of EOs and PEs to injured skin. 

## 2. Injured Skin: Microbiology

Healthy skin has its own microbiota that comprises millions of bacteria, fungi, and viruses. The main bacterial communities found on the skin belong to phyla Actinobacteria, Firmicutes, Bacteroidetes, and Proteobacteria [10], specifically to the genera *Staphylococcus*, *Propionibacterium*, *Corynebacterium*, *Streptococcus*, and *Pseudomonas* [10,11]. Skin also has a community of eukaryotic organisms formed by mites from genus *Demodex* and yeasts belonging to the genera *Malassezia* (main component of the fungal skin microbiome), *Cryptococcus*, *Rhodotorula*, and *Candida* [12]. Bacteriophages are the predominant viruses found on the skin; Densovirus, Alphapapillomavirus, Human papillomavirus, Merkel cell polyomavirus, Molluscum contagiosum virus, Polyomavirus HPyV7, Polyomavirus, HpyV6 RD114 retrovirus, and Simian virus are also present [13]. Skin microbiota protects the organism from pathogen invasion and regulates the local pH; these microorganisms respond rapidly to sudden environmental changes [14]. 

**Figure 1:**
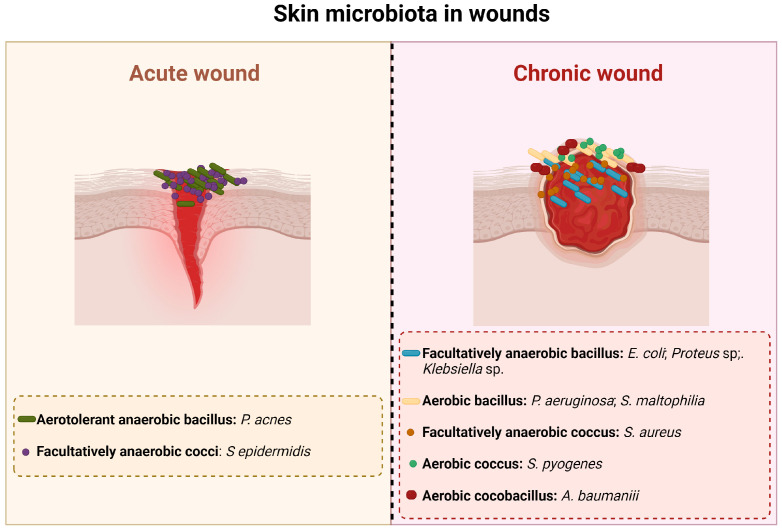
Main bacterial species present in acute and chronic wounds.

When the skin is injured, microorganisms of the normal skin flora and exogenous bacteria and fungi can penetrate it and gain access to the underlying tissues, thus having optimal conditions to colonize [15]. Based on the state of the infection and the replication cycle of the microorganisms, a wound is classified as being contaminated, colonized, locally infected, and/or spreading invasive infection [16]. So, as a result, acute and chronic wounds have different microbiota that are summarized in Figure 1.

An infection at a wound site begins with contamination. Contamination occurs due to the existence of non-replicating bacteria [15] that are part of the resident skin microbiota and/or come from the environment (transient microbiota). All chronic wounds present some level of contamination [17]. Colonization alone does not trigger a host response and thus does not delay the healing process [15,17,18]. The majority of microorganisms present in this phase are part of the normal skin flora, such as *Staphylococcus epidermidis* (*S. epidermidis*) and other coagulase-negative bacteria like *Staphylococcus* spp., *Corynebacterium* spp., *Brevibacterium* spp., *Propionibacterium acnes*, and *Pityrosporum* spp. Acute colonization is a transition state between colonization and invasive infection [18]; this phase is characterized by a moderate local reaction that is a result of the active bacterial replication [15]. Although the appearance of the wound in this stage is unhealthy, there is no microbial invasion of the tissues and most of the clinical signs of infection are absent; the only sign that is present is delayed healing, which is due to the increased bacterial concentration [15,18]. 

A wound infection occurs when microorganisms multiply and invade the surface of the wound and the deeper, healthy viable tissue on the periphery of the wound, triggering an immune response [15,17]. The first bacteria that appear on an infected wound are *Staphylococcus aureus* (*S. aureus*), Beta-hemolytic *Streptococcus* (*Streptococcus pyogenes*, *Streptococcus agalactiae*), *Escherichia coli* (*E. coli*), *Proteus*, *Klebsiella*, *Pseudomonas*, *Acinetobacter*, and *Stenotrophomonas* (*Xanthomonas*) [17]. After four or more weeks of infection, the wound is colonized by Gram-negative rods such as *Proteus*, *E. coli*, and *Klebsiella* [17]. These bacteria can penetrate the deeper layers of the skin and cause significant damage to the tissues [15,19]. As the infection progresses, anaerobic bacteria outnumber the aerobic microorganisms. Thus, in long-term chronic wounds, *Pseudomonas*, *Acinetobacter*, and *Stenotrophomonas* are commonly found [17]. The microbial invasion of the healthy tissues triggers local and systemic host reactions that manifest as purulent expulsion, spreading erythema, or symptomatic cellulitis [15]. 

The occurrence of biofilms is an important characteristic of infected wounds [16]. Bacteria living in a biofilm show changes in their phenotypes that result in alterations in virulence factors’ production in response to signaling molecules produced by other organisms in the biofilm. They also have more sessile growth and slower metabolic rates [18]. A mature biofilm confers a protective environment for the microorganisms, increasing the resistance to conventional antibiotics and shielding bacteria from the phagocytic activity of the polymorphonuclear neutrophils [16]. The existence of biofilms may explain why chronic ulcers do not heal easily [16]. 

## 3. Wound Healing

**Figure 2:**
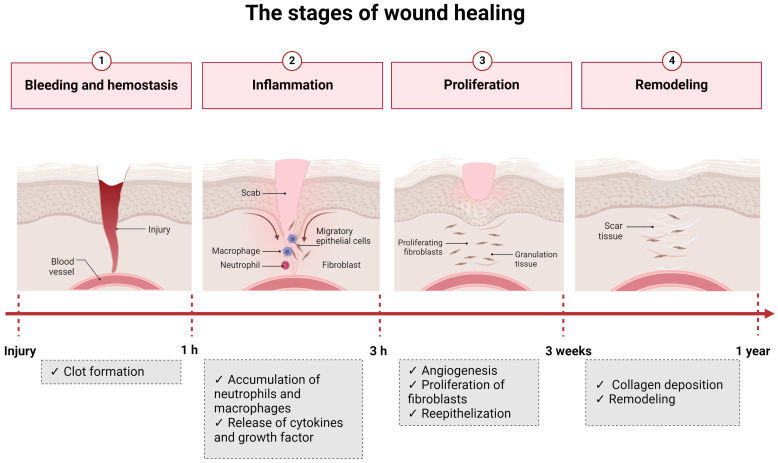
The four stages of wound healing.

The wound healing process can be divided into four stages: hemostasis, inflammation, proliferation, and remodeling [5,8] (Figure 2). 

**Figure 3:**
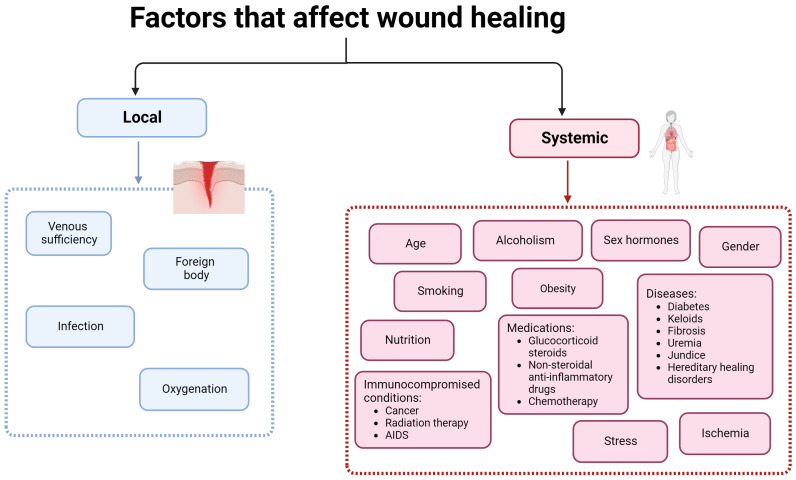
Factors that affect wound healing.

Hemostasis consists of the organism’s immediate response to an injury and aims to stop the blood loss. This phase is mediated by platelets that create blood clots [5,8]. The next stage, inflammation, begins 24 h after the injury and has a duration of 4 to 6 days. Neutrophils and macrophages are the cells responsible for this step and eliminate foreign particles and tissue debris from the wound. In this stage, cytokines and enzymes are released to stimulate fibroblasts and myofibroblasts. The exudate confers the necessary moisture for recovery to the wound [5,8]. The proliferation phase is characterized by the re-epithelization and formation of new granulation tissue that begins to fill the wounded area. This stage has a duration of 4 to 21 days [5,8]. Lastly, in the remodeling phase, a tight 3D network is formed through collagen-based crosslinking, increasing the tensile strength of the new tissue [5,8]. There are a series of factors that can influence the wound healing process. They can be divided into local and systemic and are listed in Figure 3.

Systemic factors such as age, sex hormones/gender, stress, alcohol consumption, smoking, obesity, nutrition, ischemia immunocompromised conditions, and some medications have an important impact on wound healing [4]. Increased age delays wound healing but does not affect the quality of the process. The delay of the wound healing process in aged people is due to the alteration of the inflammatory response, re-epithelization, collagen synthesis, and angiogenesis [4,16]. Sex hormones also affect wound healing, resulting in significant differences between males and females. Female estrogen hormones regulate a variety of genes associated with regeneration, matrix production, protease inhibition, epidermal function, and genes related to inflammation [4,16]. Furthermore, estrogen is known to improve age-related impairment in the healing process, while androgen affects it negatively [20].

Stress has a huge impact on human health and affects the wound healing process by delaying it. Stressful conditions lead to an up-regulation of glucocorticoids, reducing the levels of pro-inflammatory cytokines and chemo-attractants, which are both necessary in the inflammatory phase. Additionally, glucocorticoids influence immune cells by suppressing their differentiation and proliferation, reducing the production of cell adhesion molecules and regulating gene transcription [4]. 

Several diseases also affect the wound healing process; diabetes, in particular, is a condition in which the affected individuals show delayed and impaired wound healing. Furthermore, diabetic individuals can suffer from diabetic foot ulcer, which is followed by hypoxia, leading to insufficient angiogenesis, enhancing early inflammatory response, and increasing the levels of oxygen radicals. Additionally, hyperglycemia increases the levels of reactive oxygen species (ROS), increasing the effect of oxidative stress [4].

Obesity is a well-known risk factor for a series of diseases, such as coronary heart disease, type 2 diabetes, cancer, hypertension, dyslipidemia, stroke, sleep apnea, and respiratory problems, and it also affects wound healing [16]. Obese individuals frequently suffer from wound complications, like infections, dehiscence, hematoma and seroma formation, pressure ulcers, and venous ulcers [21]. Individuals who undergo bariatric and non-bariatric surgeries have high infection rates at the surgical site due to relative hypoperfusion and ischemia that occur in subcutaneous adipose tissue, resulting in a decreased delivery of antibiotics to the site [4,16].

Alcoholism and smoking are also two risk factors for impaired wound healing. Alcohol exposure increases the vulnerability of a wound to infection because it interferes with defense mechanisms [4]. Smoking negatively affects wound healing, too; smokers present delayed wound healing and increased risk of infection, wound rupture, anastomotic leakage, flap necrosis, and epidermolysis [16]. 

Local factors have a direct impact on the wound healing process, and oxygen is a particularly important one. Oxygen is crucial for cell metabolism, energy production, and is vital in all steps of the wound healing process. It prevents the infection of wounds, induces angiogenesis, increases keratinocyte differentiation, migration and re-epithelization, enhances fibroblast proliferation and synthesis of collagen, and promotes wound contraction [4]. Additionally, the production of the superoxide anion, for the oxidative killing of pathogens, is dependent on oxygen levels. The rupture of blood vessels in the wound site decreases the levels of oxygen, leading to hypoxia. Temporary hypoxia helps the wound healing process because it induces macrophages, fibroblasts, and keratinocytes to produce cytokines and growth factors crucial for cell proliferation and migration, chemotaxis, and angiogenesis. However, chronic hypoxia delays the healing process because this phenomenon leads to an increase in the concentration of ROS (produced during normal oxygenation), which is prejudicial for damaged tissues [4,16]. Another important factor that affects the wound healing process, delaying it, is the existence of infections [22], as discussed in detail in the previous section. 

## 4. Wound Healing: A Brief History and Current Treatments

Every wound, whether it is acute or chronic, needs to be treated. The process of wound healing requires dressings and bandages [23]. A dressing is a formulation designed to be in contact with the wound, whereas a bandage is a structure that holds the dressing in its right place [23]. Wound dressings function as barriers that shield the wound and prevent contamination and infection. The use of an appropriate dressing is extremely important to ensure adequate wound protection and accelerate the healing process [24].

Historically, the ancient-known record of wound healing was found in clay tablets in Mesopotamia and dates to 2500 BCE. In this medical record, the three steps of wound healing are described for the first time: washing of the wounds, making of the plasters (wound dressings), and bandaging of the wounds [23,25]. Mesopotamians washed wounds with water or milk and then applied honey or resin as dressings [26]. 

In ancient Egypt, the wound healing process had a spiritual basis; as an open wound was considered a possible entry point for malicious creatures, it needed to be treated with a repellent to safeguard the integrity of the spiritual vessel. Usually, feces from donkeys were used as they possessed some antibiotic substances and proteins, such as trypsin, which help the healing process [26]. Other wound healing treatments consisted of the use of adhesive tape and gauze to close a clean wound and to cover the wound with fresh meat on the first day, followed by treatment with astringents, herbs, and honey [26].

Ancient Greeks distinguished between acute or “fresh” wounds as well as non-healing or chronic wounds and used clean boiled water, vinegar, and wine to wash them. Hippocrates (460-370 BCE) washed wounds with wine or vinegar and then treated them with honey, oil, and wine [25,26]. Boiled wool in water or wine was used as a bandage [23].

Traditional Chinese medicine is similar to other ancient medicines and has not changed much over the centuries. It uses bronze instruments, green tea, licorice, soaked mushrooms, anesthetics, soporific drugs, antiseptics, and other herbal powders to promote tissue granulation, aid in debridement, and help to avoid infection. Gauze and silk have been used as bandages [26,27].

In the 19th century, the discovery of antibiotics allowed us to control infections and helped to decrease mortality rates. The discovery of the antiseptic technique was a massive progress in wound healing [23,25]. The advent of modern wound healing occurred in the 20th century. The production of occlusive dressings that protect and provide a moist environment to the wounds began. These new dressings enabled a faster re-epithelization and collagen synthesis, promoted angiogenesis, and decreased wound infection [23]. Nowadays, there are more than 5000 wound care products [25]. Table 1 lists the types of wound dressings available and their advantages and disadvantages. 

An ideal dressing must provide a moist environment to reduce the risk of scar formation, remove excessive exudates, favor the epithelization and cell migration into the wound, improve autolytic debridement, and act as a barrier against external threats, inhibiting the growth of pathogenic fungi and bacteria [28]. It may also show mechanical stability during application, wearing, and removal, while maintaining an elastic texture to adapt to the wound and some flexibility that allows the patient to move [29,30]. An appropriate wound dressing may also be easy to use, non-allergic, non-toxic, cost-affordable, and assure rapid healing [4,30]. The main purpose of wound dressings is their ability to accelerate the healing process. Therefore, the newest formulations that possess improved biocompatibility and humidity retention can improve the hypoxic environment, thus speeding up the process [31]. 

Chronic wounds pose an additional challenge regarding wound healing because they produce large volumes of exudates, requiring frequent dressing changes. Thus, the dressings used in these wounds must present low adherence to protect the newly formed tissue from destruction during dressing removal [29]. Additionally, these wounds require an active intervention in the healing process, with dressings that allow the release of drugs and/or dressings that can to be incorporated in the cells [29].

**Table 1:** Types of wound dressings.

Type of Dressing	Formulation	Advantages	Disadvantages	Commercially Available Products	References
Inert/passive	Gauzes	Manufactured in the form of bandages, sponges, plasters, and stockings. Possess high porosity, make thermal isolation available, and sustain a human environment at the wound site. Sponges can be applied directly to the surface of suppurating wounds. Inexpensive.	Can stick to wounds and disrupt the wound bed when removed. Suitable mostly for minor wounds. Sponges are not ideal for third-degree burns or wounds with desiccated eschar because of the lack of mechanical resistance.	Curity, Vaseline Gauze, Xeroform, Multisorb, Urgotul SSD/S.Ag	[15,32]
Bioactive	Hydrocolloids	Semi-permeable formulations that can comprise hydroactive particles that swell with exudates or form a gel. Can be easily detached from wounds with the help of saline or sterilized water. Painless dressings (highly recommended for pediatrics wound care management).	Contraindicated for heavily draining wounds, infected wounds, arterial ulcers, third-degree burns, and exposed tendons/fascia. Can be cytotoxic. Can have a disagreeable odor and an acid pH at the application site. Present a low mechanical strength.	DouDERM, Granuflex, Comfeel, Tegasorb	[8,15]
	Alginates	Highly absorbent and hemostatic. Appropriate for exudating wounds. Useful in the debridement of sloughing wounds.	Limited use on low exudating wounds because they can cause dryness and scabbing. Need to be changed daily.	Algisite, Kaltostat, Sorbsan, Tegagen, SeaSorb, PolyMem	[15,32]
	Collagens	Stimulate the formation and setting of newly formed collagen in wounds. Absorb large amounts of exudates and maintain a humid environment in the wounds. Shield the wound against mechanical trauma and infections. Easy to apply, non-immunogenic, and non-pyrogenic. Exist in the form of pads, gels, films, membranes, and particles.	Application not recommended in wounds with necrosis and third-degree burns. Needs a secondary dressing.	Puracol Plus, Triple Helix Collagen, Cutimed Epiona Sterile, BIOSTEP	[18,33]
	Hydrofibers	Highly absorbent fibers form a gel when in contact with wound exudates. Vertical wicking of the exudate helps to reduce the wound’s maceration. Favors autolytic debridement. Only needs to be replaced when the dressing is saturated.	Some fluid absorption is required for pH control, but the absorption of an excessive amount of fluid can cause undesirable swelling of the wound dressing, resulting in distension and loss of adhesion. Should not be used on dry wounds because they can produce a fibrous residue. In mildly exudating wounds, the dressing may need to be soaked in sterile water or saline solution before removal to avoid trauma.	Aquacel	[15,34]
Interactive	Hydrogels	Rehydrates dry wounds. Keeps the wound moist whilst absorbing extensive exudate. Easy removal. Permeable to metabolites. Non-irritant. Non-reactive with biological tissues. Pain reduction due to cooling and soothing effects on the skin. Favors autolytic debridement without damage to the epithelial cells or granulation.	Can cause over-hydration. Possess weak mechanical properties thus needing a secondary dressing. Should not be used in highly exudating wounds.	Carrasyn, Curagel, Nu-Gel, Purilon, Restore, SAF-gel, XCell	[15,29,32,34]
	Semi-permeable films	Semi-permeable. Allow inspection of the wound without dressing removal due to its transparency. Permeable to water vapor, O_2_, and CO_2_. Highly elastic. Adapts easily to the patient’s body. Reduce pain. Serve as a barrier from external contamination.	Not appropriate for moderately to highly exuding wounds. May cause maceration of the surrounding skin. May damage fragile skin.	Opsite, Tegaderm, Biooclusive, Polyskin, Blisterfilm, Cutifilm, Flexigrid	[15,23,32,34]
	Semi-permeable foams	Soft. Can be hydrophobic or hydrophilic. Can absorb large amounts of exudates depending on the wound thickness. Provide thermal insulation.	Not adequate for the treatment of dry wounds, necrotic wounds, and eschars because they can cause dryness and scabbing. People with fragile skin may require special care. Can require a retention product.	Allevyn, Lyofoam, Tielle, Curafoam, Mepilex, Permafoam, Tegafoam,	[15,23,34]
Skin substitutes		Adequate for the treatment of chronic, non-healing ulcers. Provide temporary or permanent wound closure. Reduce healing time and post-operative contracture. Decrease morbidity from invasive treatments. Reduced scaring. Reduce pain levels and nursing requirements.	Expensive. Reduced shelf life. Some skin substitutes possess a risk of transmission of infectious diseases. Some products present a risk of donor rejection.	Epicel^®^, Laserskin^®^, TransCyte^®^, Dermagraft^®^, AlloDerm^®^, Strattice^®^, Biobrane^®^, Integra^®^ Dermal Regeneration Template, Apligraft^®^, Graftskin^®^, OrCell^®^, Graftjacket^®^, PermaDerm^®^	[35]

## 5. The Role of Essential Oils and Plant Extracts in the Wound Healing Process

Natural compounds of plant origin have been used by humanity for centuries to treat wounds [26]. Amongst these, essential oils (EOs) and plant extracts (PEs) have recently attracted the attention of the scientific community [36]. 

EOs are secondary metabolites synthesized by several plant organs, such as leaves, seeds, bark, twigs, and roots [15]. They have antioxidant, anti-inflammatory, anti-allergic, antimicrobial, and regenerative properties [15], which makes them useful in the wound healing process. PEs are acquired from natural plants and possess antioxidant, antimicrobial, and immune response mediator activities [37,38]. Additionally, they are effective at low concentrations, cost-effective, easy to apply, and their toxicity levels are low [38]. Several solvents can be used for the obtention of PEs (Table 2). Polar solvents (acetone, ethanol, and methanol), except water, are usually able to extract a wide range of phytochemicals (phenols, flavonoids, etc.) from plants. As such, these extracts present greater antimicrobial activity when compared to the extracts obtained from non-polar solvents (hexane, ethyl-acetate, etc.) [39]. 

Both can be used in the treatment of wounds because they can be involved in all stages of the wound healing process. These substances can interact at the intracellular level in the modulation of ROS generation, thus increasing the response of immune cells, which leads to a decrease in the inflammatory state and acceleration of tissue regeneration. Moreover, EOs and PEs prevent the deterioration of granulation tissue and help the proper functioning of growth factors and extracellular matrix components, thus contributing to the normal progress of the healing process [40]. 

**Figure 4:**
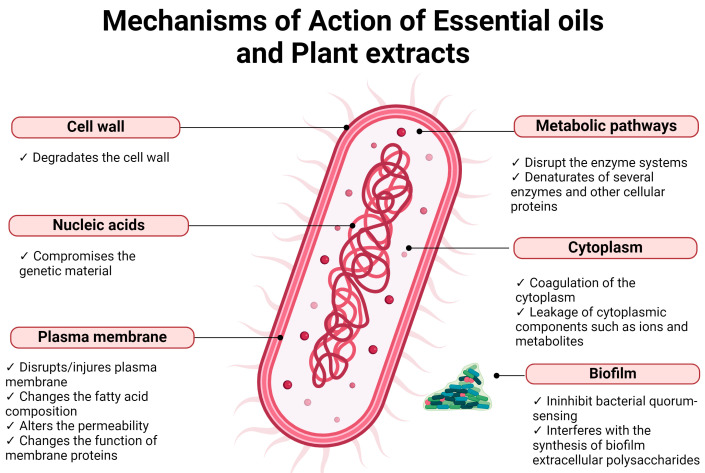
Mechanisms of action of EOs and PEs on bacterial cells.

The occurrence of infections is an important factor that affects the wound healing process. Infected wounds frequently need the use of antimicrobial agents for their treatment. However, the increase in antimicrobial-resistant microorganisms requires the use of new agents, particularly those of natural origin [15]. EOs and PEs display antimicrobial activity against several microorganisms, including the ones that are more commonly found on infected wounds (Table 2). The antimicrobial potential of these substances results from the effect of different molecules on different cell targets [41] (Figure 4).

The cell membrane of bacteria is one of the targets of EOs. They damage the outer membrane of Gram-negative bacteria, increasing the permeability of the cytoplasmatic membrane, which leads to the leakage of ATP, changes the fatty acid composition, disrupts the enzyme systems, and compromises genetic material [42,43]. However, in some Gram-negative bacteria, the existence of an external capsule can limit the entry of EOs into the cell [44]. Gram-positive bacteria are usually more sensitive to EOs than Gram-negative bacteria, due to the amount of peptidoglycan (90–95%) in their cell wall, which allows for the EOs to penetrate the cell wall more easily, damaging the cell membrane and causing alterations in its structure and functionality [44]. One characteristic of EOs that explains their capacity to affect the membrane of bacterial cells is their hydrophobicity, which enables easy diffusion through the lipid bilayer and alters the permeability and function of membrane proteins [44]. Additionally, EOs can cause coagulation of the cytoplasm, leakage of cytoplasmic components such as ions and metabolites, reduction in the proton motive force and the intracellular ATP pool by decreasing the ATP synthesis, and denaturation of several enzymes and other cellular proteins [44,45]. Some studies also suggest that EOs can inhibit bacterial quorum sensing by interfering with quorum-sensing-responsible molecules produced by bacteria. This results in the reduction of proteolytic activity, biofilm formation, and swarming motility [44,46].

The main mechanism of action of PEs in bacterial cells seems to be the rupture of the cell membrane [39,47,48,49,50,51], which leads to the leakage of cell content [48,51] and subsequent death. PEs also cause the depletion/leakage of intracellular ATP [51,52] and disrupt cell metabolism by destroying proteins and/or inhibiting their synthesis [50]. In bacteria that have the ability to form biofilm, such as *S. aureus* and *S. epidermidis*, PEs can suppress its formation because they interfere with the synthesis of biofilm extracellular polysaccharides [53].

**Table 2:** Antibacterial activity of some EOs and PEs for bacterial species commonly found on infected wounds.

		Minimum Inhibitory Concentrations (%)							References
Essential oils		***Acinetobacter* sp.**	***E. coli***	***K. pneumoniae***	***P. aeruginosa***	***P. vulgaris***	***S. aureus***	***S. epidermidis***	
	*Arborvitae* sp.			0.125	0.25	0.125			[54]
	*Cassia* sp.			0.125	0.125	0.125			[54]
	*Cinnamomum zeylanicum* (Cinnamon)	0.8		0.2		0.8	0.05	0.1	[55]
	*Cymbopogan citratus* (Lemongrass)		0.06	0.25	0.25	0.25			[54,55]
	*Eucalyptus* sp. (eucalyptus)			1.25	2.5	1.25			[54]
	*Lavandula officinalis* (Lavender)		0.2				0.1		[55]
	*Melaleuca alternifolia* (Tea tree)			0.125	0.5	0.125			[54]
	*Pimenta dioica* (Jamaica pepper)	0.8		0.4			0.1	0.1	[55]
	*Piper betle* (Betel)	0.8		0.4		0.4	0.05	0.05	[55]
	*Psiadia arguta*	1.6					0.05	0.025	[55]
	*Psiadia terebinthina*	1.6		0.4		0.8	0.05	0.025	[55]
	*Origanum vulgarae* (Oregano)		0.115	0.05	0.125	0.05	0.029		[54,56]
	*Rosmarinus officinalis* (Rosemary)		0.256						[57]
	*Salvia officinalis* (Sage)		>0.256						[57]
	*Satureja montana* (Winter savory)				2.33				[55]
	*Syzygium aromaticum* (Clove)			0.125	0.5	0.125			[54]
	*Thymus vulgaris* (Thyme)		0.064	0.05	0.125	0.05			[54,57]
Plant extracts	*Acacia nilotica* ^1^		0.312						[47]
	*Bauhinia kockiana* ^2^						0.00625		[48]
	*Cistus salviifolius* ^3^						0.00807		[49]
	*Cytinus hypocistis* ^1^			>0.05	>0.05		0.0125	0.025	[53]
	*Cytinus ruber* ^1^			>0.05	>0.05		0.0125	0.025	[53]
	*Phaseolus vulgaris* ^4^		0.0512	0.0512	0.0512				[58]
	*Punica granatum* ^3^						0.005167		[49]
	*Quercus variabilis* ^1^						0.0625		[50]
	*Smilax china* ^1^		0.0195				0.0195		[51]
	*Theobroma cacao* ^4^		0.0064	0.0064	0.1024				[58]
	*Triumfetta welwitschii* ^1^			0.01	0.01				[39]

Types of solvents used in the preparation of the extracts: 1—ethanol; 2—ethyl acetate; 3—water; 4—methanol.

## 6. Formulations Incorporated with EOs and PEs

Natural products, such as EOs and PEs, present antioxidant, anti-inflammatory, antimicrobial, and analgesic properties [59], making them good alternatives to the current drugs used in the treatment of wounds. However, despite these properties, their therapeutic potential and use are limited due to their lack of targeting capacity and poor bioavailability [28,60]. Therefore, finding new strategies able to deliver these substances to wounds and to overcome these problems is important. Formulations with drug-releasing capacities allow us to reduce antimicrobial doses, decrease the risk of systemic toxicity, and deliver antimicrobial agents to wounds with poor blood circulation [61], thus being a potential vehicle for the delivery of PEs and EOs to wounds. 

### 6.1. Films 

The use of films for wound treatment dates back to 1945, when cellophane was used to treat burns in World War II prisoners [62]. Nowadays, polymeric films are thin, flexible, non-toxic, biocompatible, and biodegradable membranes that are adhesive on one side [62,63] and are usually prepared by solvent casting, which is a low-cost method with easy manufacturing [63]. This technique requires the preparation of film-forming solutions, composed of natural or synthetic polymer(s), plasticizing agents, and, in some cases, crosslinking substances [64]. The solutions are then poured into molds and left to dry completely until the films are formed [63]. 

Natural polymers, such as alginate, chitosan, keratin, starch, gelatin, and cellulose, are biocompatible and biodegradable, have regenerative and adhesive properties, and are inert [65,66]. However, they are susceptible to microbial contamination and have poor mechanical properties [65]. Synthetic polymers, such as polyvinyl alcohol (PVA), polyacrylic acid (PAA), poly-ε-caprolactone (PCL), polyethylene glycol (PEG), polyvinylpyrrolidone (PVP), and polylactic acid (PLA), have better mechanical properties (strength, flexibility, structure, and higher degree of polymerization) than natural polymers. However, they have low biocompatibility (that may cause immunological reactions, which can result in the rejection of the film), low adherence to wounds, and lower absorption and permeability [65]. 

Some characteristics that can be assigned to polymeric films and explain their usefulness in the wound healing process are (a) flexibility and elasticity, which allow for an easy adjustment to the body and can then be used in difficult areas, such as joints; (b) impermeability to water and permeability to gas, allowing for some moisture evaporation; (c) ability to be a barrier between the wound and the environment, thus avoiding external contamination; (d) transparency, which allows the inspection of the wound without removing the dressing, therefore avoiding constant dressing changes; (e) ability to be used for direct drug delivery to the injured skin; and (f) ease of application [24,63]. However, since films are formulations with low porosity, they cannot be used in high exudate wounds because they are not able to absorb high quantities of biological fluids [24].

Films can be helpful in all phases of the healing process. During the hemostasis stage, polymeric films act as a barrier to prevent blood loss and work as a scaffold for immune cells, cytokines, and growth factors. Because polymeric films are drug carriers, they can be used in the inflammation stage to deliver antibiotics and anti-inflammatory drugs to the wound, effectively controlling infections and inflammation and enhancing the body’s natural defense mechanisms. In the proliferation stage, these formulations can stimulate the formation of granulation tissue, deposition of collagen, angiogenesis, re-epithelization, and wound contraction. Finally, in the remodeling stage, polymeric films can help in the transition from collagen type III to collagen type I and in the reorganization of collagen fibers [65,67]. 

EOs and PEs—due to their antimicrobial, antioxidant, and anti-inflammatory potential, as well as their ability to speed up closure rate and enhance collagen deposition and fibroblasts proliferation [66]—can be added to polymeric films, increasing their healing potential. The incorporation of EOs and PEs into films is usually performed with emulsification or homogenization techniques, changing its functionality [68,69], but maintaining the healing properties of these substances [66]. Yet, the possibility of using films with EOs and PEs as wound dressing materials only began to be explored since 2010 [66]. 

Table 3 lists some studies involving films incorporated with EOs and PEs, emphasizing their antimicrobial properties. The antimicrobial activity against pathogenic bacteria commonly found in wounds is normally evaluated via diffusion assays and is usually higher for Gram-positive bacteria than for Gram-negative bacteria [70,71].

In addition to their antimicrobial capacity, films incorporated with PEs and EOs have other properties that make them interesting for wound treatment. In a study regarding the production and characterization of chitosan films with tea tree oil [75], the authors showed that their formulations had fluid absorption and blood clotting abilities, which are important characteristics in a wound dressing. Several studies also report that polymeric films incorporated with PEs and EOs can promote fibroblast [71,75,82,85] and keratinocyte [86] proliferation, maintaining their viability. Furthermore, films with PEs and EOs can promote fibroblast migration [82,85,87], enhancing the wound healing process. The authors in [71] produced chitosan films with *Hypericum perforatum* EOs and verified that these formulations were good surfaces for cell attachment. Moreover, various studies mention that films with EOs and PEs are biocompatible [78,82,83,86,87], presenting low cytotoxicity [79]. 

Nonetheless, despite the promising results of antimicrobial films incorporated with EOs and PEs, their activity in vivo is still poorly understood [80,81].

### 6.2. Hydrogels 

Hydrogels (HGs) are hydrophilic, three-dimensional matrices made of water-insoluble polymers [29,88]. These formulations have a water content of around 90% and can swell with water without dissolving [34,89]. The word “hydrogel” was mentioned in the literature for the first time in 1894, and it referred to a colloidal gel of inorganic salts [88,90]. However, it was only in 1960 that Wichterle and Lím [91] developed hydrogels with characteristics that are currently assigned to these formulations. Since then, the number of studies on hydrogels has grown exponentially, especially after the 1990s [88,90]. The development of hydrogels occurred in three phases, as described by Buwalda et al. [92]. In phase 1, the hydrogels consisted of simple formulations with good mechanical and swelling properties and were produced with the method proposed by Wichterle and Lím (1960) [91]. The second phase, initiated in the 1970s, comprised hydrogels that were able to respond to stimuli, like pH and temperature, inducing a specific response. Phase 3 consisted in the production of hydrogels with supramolecular complexes, with good biocompatibility and versatility. “Smart hydrogels”, formulations with a wide number of adaptable properties, originated at this stage [88,92]. 

Hydrogels are considered one of the most promising dressings for wound care [29]. Unlike traditional dressings that can only cover wounds, maintain adequate gas exchange, and adhere strongly to the wound, causing pain and additional lesions when changed [29], hydrogel-based dressings have excellent biocompatibility, high moisture resistance, and the ability to activate immune cells, thus fulfilling important requirements for an ideal wound dressing [93]. Additionally, HGs have many more effects on the wound healing process, such as enhancement of skin regeneration; development of skin appendages; acceleration of collagen secretion and deposition; induction of fibroblasts and keratinocytes migration; formation of capillary vessels; stimulation of wound healing; acceleration of the recruitment of endothelial cells and cell progenitors into the wound area; acceleration of angiogenesis; normalization of pro-inflammatory cytokines; progress in wound contraction; stimulation of early infiltration and degradation of inflammatory cells; promotion of neovascularization; increase in tissue granulation; reduction of fluid secretion; sustained release of therapeutic substances; creation of thinner scrabs; facilitation of dressing removal; restoration of skin function; effect on gene regulation; increase in vessel density; attenuation of scar formation; acceleration of epidermal differentiation; regulation of protein levels; and development of hair follicles and sebaceous glands [94].

As wounds are prone to infection via pathogenic microbes, it is necessary to have a dressing that acts as a barrier against infectious microorganisms, inhibits their growth, and stimulates skin healing [95]. HGs with antimicrobial activity can be obtained via two possible methods: (1) the HG itself has antimicrobial activity or (2) the HG is loaded with antimicrobial substances that are incorporated by physical or chemical reactions into the gel [96]. Since PEs and EOs possess antimicrobial activity against bacteria present in infected wounds, their incorporation into HGs gives them the ability to enhance wound healing. Some studies point to the possibility of using HGs with PEs and EOs for the management of chronic wounds (Table 4). The selected studies show the antimicrobial activity of hydrogel formulations against the bacteria responsible for wound infections, which is normally determined using diffusion assays.

Another important characteristic of a wound dressing is its biocompatibility. Several studies [97,98,103,106,108,109,110] demonstrated that HGs incorporated with PEs and EOs are biocompatible with minimal or no cytotoxicity. These formulations are also able to maintain fibroblast viability [101,104,106], while inducing their migration [97,109,110] and proliferation [101,104,106]. Hemocompatibility is an essential feature of a wound dressing because it may help in the healing process without causing blood toxicity. Some studies indicate that hydrogels with PEs and EOs are compatible with blood components and do not induce significant hemolysis [98,106,110,111]. 

Due to the proven healing potential of these formulations, several authors proceeded with in vivo studies [110,112,113]; usually performed on mice models, these works report that hydrogels incorporated with PEs and EOs—due to the phytochemicals present in these compounds—accelerate the wound healing process [110,113,114], when compared to traditional treatments (such as Betadine), with minimal scar formation [112]. These formulations also contribute to the maintenance of a moist environment in the wound, which enhances fibroblast and keratinocyte proliferation and promotes the deposition of collagen [110,111]. However, despite the promising results of antimicrobial HGs incorporated with EOs and PEs, their application on humans is still scarce [115], and more studies are required for these formulations to become a real alternative to conventional wound dressings.

## 7. Conclusions

Skin is constantly exposed to external threats, which can cause wounds. Chronic wounds, in particular, are a major health problem that affects millions of people worldwide and results in costs comprising billions of dollars for national health services. These wounds are usually prone to infection, which delays their treatment even further. Nowadays, various wound dressings can be used to treat them, some of which are incorporated with antimicrobial agents. However, the number of microbes resistant to these substances is rising and, as such, there is a surge of new and natural alternatives. 

In this work, the use of films and HGs for the delivery of EOs and PEs to the skin for wound treatment was discussed. The antimicrobial activity of EOs and PEs against the main bacterial species present in wounds is documented by several works and varies depending on the essential oil or extract used. Some studies also show that HGs and films incorporated with PEs and EOs have antimicrobial activity, promote the viability of skin cells (fibroblasts and keratinocytes), promote fibroblast migration, and are non-toxic and biocompatible. Moreover, research indicates that HGs with EOs and PEs accelerate the wound healing process in animal models. So, films and hydrogels incorporated with EOs and PEs may be considered promising substitutes to the current treatments for wound healing. However, for these formulations to constitute an alternative to the current wound dressings, more human trials are required. 

Regarding the use of films incorporated with EOs and PEs, their activity in vivo is still poorly understood and, as such, more studies are needed to further elucidate their action mechanisms for them to be used as effective wound dressings. 

## Figures and Tables

**Table 3:** Films incorporated with EOs (a) and PEs (b) for wound healing applications.

**(a) Films Prepared by Solvent Casting Incorporated with EOs**							
**Film Characteristics**			**Antibacterial Activity**				
**Polymer**	**Plasticizer**	**Essential Oil**	**Experimental Method**	**Studied Species**	**Antibacterial Activity Values**	**Intended Application**	**Reference**
Alginate	Glycerol	Cinnamon	Disc diffusion assay	*E. coli*	12 mm	Wound dressing	[72]
		Lavender	Disc diffusion assay	*E. coli*	2 mm	Wound dressing	[72]
		Tea tree	Disc diffusion assay	*E. coli*	2 mm	Wound dressing	[72]
		Peppermint	Disc diffusion assay	*E. coli*	2 mm	Wound dressing	[72]
		Lemongrass	Disc diffusion assay	*E. coli*	3 mm	Wound dressing	[72]
Chitosan	Poly-vinyl alcohol	Cinnamon	Time-kill kinetics	*S. aureus* *P. aeruginosa*	Inhibition at 6 h No inhibition	--------------	[70]
		Clove	Time-kill kinetics	*S. aureus* *P. aeruginosa*	Inhibition at 24 h No inhibition	--------------	[70]
	Glycerol	Eucalyptus	Disc diffusion assay	*E. coli* *S. aureus* *P. aeruginosa*	153.37 mm^2^ 61.35 mm^2^ 118.29 mm^2^	--------------	[73]
		Clove bud	Disc diffusion assay	*E. coli* *S. aureus*	5 mm 20 mm	--------------	[74]
		Cinnamon	Disc diffusion assay	*E. coli* *S. aureus*	10 mm 30 mm	--------------	[74]
		Tea tree	Turbidimetric method	*E. coli* *S. aureus*	No inhibition A significant difference in optical density	Wound healing	[75]
		Thyme	Agar diffusion assay	*E. coli* *K. pneumxoniae* *S. aureus* *P. aeruginosa*	17 mm 19 mm 16 mm 16 mm	Wound healing	[76]
		Clove	Disc diffusion assay	*E. coli* *S. aureus*	8 mm 9 mm	Wound dressing	[77]
		Tea tree	Disc diffusion assay	*E. coli* *S. aureus*	9 mm 6 mm	Wound dressing	[77]
		*Hypericum perforatum*	Agar diffusion assay	*E. coli* *S. aureus*	2.9 ± 0.1 cm 1.97 ± 0.05 cm	Wound dressing	[71]
Chitosan/collagen		Lemongrass	Colony count method (inhibition percentage)	*E. coli* *S. aureus*	99.8% 99.9%	--------------	[78]
**(b) Films Prepared by Solvent Casting Incorporated with PEs**							
**Antibacterial Activity**							
**Polymer(s)**	**Plasticizer**	**Plant Extract**	**Experimental Method**	**Studied Species**	**Antibacterial Activity Values**	**Intended Application**	**Reference**
Chitosan	Glycerol	*Mimosa tenuiflora*	Turbidimetry assay	*E. coli* *Micrococcus luteus*	24% 95%	Skin regeneration	[79]
O-carboxymethyl chitosan	Glycerol	*Mimosa tenuiflora*	Turbidimetry assay	*E. coli* *Micrococcus luteus*	22% 17%	Skin regeneration	[80]
Poly-vinyl alcohol		*Aloe vera*	Disc diffusion assay	*E. coli* *P. aeruginosa*	16 mm 16 mm	Coating of surgical sutures	[81]
Poly-vinyl alcohol/starch/polyacrylic acid	Glycerin	*Punica granatum*	Disc diffusion assay	*S. epidermidis* *MRSA*	23 mm 20 mm	Wound healing	[82]
Collagen/fibrin	Ethylene glycol	*Macrotyloma uniflorum*	Agar well diffusion assay	*B. subtilis* *S. aureus* *P. vulgaris* *E. coli*	Presents antibacterial activity against all studied species	Burn Wound dressing	[83]
Poly (Vinyl Alcohol)-Poly (Ethylene Oxide)-Carboxymethyl Cellulose		*Curcuma longa (curcumin)*	Colony count method (inhibition percentage)	*E. coli* *S. aureus*	74.7% 96%	Wound dressing	[84]
Alginate		*Hypericum perforatum*	Viable cell count	*E. coli* *S. aureus*	Presents antibacterial activity against all studied species.	Wound dressing	[85]

**Table 4:** HGs incorporated with EOs (a) and PEs (b) for wound healing applications.

**(a) HGs Incorporated with EOs**							
**Hydrogel Constitution**	**Essential Oil**	**Preparation Method**	**Antibacterial Activity**			**Intended Application**	**Reference**
			**Experimental Method**	**Studied Species**	**Antibacterial Activity Values**		
Chitosan/carboner 940	Eucalyptus	Simple blending	Turbidimetric analysis	*E. coli* *S. aureus*	46.26% 63.05%	Burn wound	[97]
Chitosan/carboner 940	Ginger	Simple blending	Turbidimetric analysis	*E. coli* *S. aureus*	18.21% 38.41%	Burn wound	[97]
Chitosan/carboner 940	Cumin	Simple blending	Turbidimetric analysis	*E. coli* *S. aureus*	22.90% 53.67%	Burn wound	[97]
Carbomer 940/chitosan	Eucalyptus	Simple blending	Turbidimetric analysis	*S. aureus*	Greater than 50%	Wound healing	[98]
Gellan gum/propylene glycol/calcium chloride	Lavender	Solvent casting ionotropic gelation	Agar well diffusion method	*E. coli* *S. aureus*	20 mm 21 mm	Wound healing	[95]
Gellan gum/propylene glycol/calcium chloride	Tea tree	Solvent casting ionotropic gelation	Agar well diffusion method	*E. coli* *S. aureus*	30 mm 31 mm	Wound healing	[95]
Gelatin/glutaraldehyde	*Eupatorium adenophorum*	Solvent casting ionotropic gelation	Disc diffusion assay	*E. coli* *S. aureus* *S. epidermidis* *B. cereus*	23 mm (circa) 29 mm (circa) 26 mm (circa) 26 mm (circa)	Wound healing	[99]
Starch/poly-vinyl alcohol/glycerin	Oregano	Solution casting	Disc diffusion assay	*E. coli* *S. aureus*	31 mm 34 mm	Wound dressing	[100]
Starch/poly-vinyl alcohol/glycerin	Tea tree	Solution casting	Disc diffusion assay	*E. coli* *S. aureus*	32 mm 35 mm	Wound dressing	[100]
Starch/poly-vinyl alcohol/glycerin	Clove	Solution casting	Disc diffusion assay	*E. coli* *S. aureus*	37 mm 39 mm	Wound dressing	[100]
κ-Carrageenan/polyethylene glycol	Thyme	Solution casting	Disc diffusion assay	*E. coli* *S. aureus*	13.8 mm 14.9 mm	Wound dressing	[101]
Gelatin/poly-vinyl alcohol/glycerol/glutaraldehyde	*Zataria multiflora*	Simple blending	Microdilution method	*P. aeruginosa* *E. coli* *S. aureus* *B. subtilis*	400 µg/mL 200 µg/mL 200 µg/mL 100 µg/mL	Wound dressing	[102]
Polyvinyl alcohol	Oregano	Freeze-thawing	Serial dilutions method	*E. coli* *S. aureus*	Complete inhibition of both bacteria	Diabetic ulcers	[103]
**(b) HGs Incorporated with PEs**							
**Hydrogel Constitution**	**Plant Extract**	**Preparation Method**	**Antibacterial Activity**			**Intended Application**	**Reference**
			**Experimental Method**	**Studied Species**	**Antibacterial Activity Values**		
Chitosan	*Hemigraphis alternata*	Freeze-drying	Viable cell method (after 24 h)	*E. coli* *S. aureus*	0.5 × 10^10^ CFU 1 × 10^10^ CFU	--------------	[104]
Chitosan/cellulose	*Calendula offcinalis*	Simple blending	Agar well diffusion method	*S. aureus* *E. coli* *P. acnes*	4 mm 2 mm 2 mm	Chronic wound	[105]
Chitosan/EDTA/β-glycerol phosphate	*Aloe vera*	Simple blending	Time-kill assay	*P. aeruginosa* *S. aureus*	Antibacterial activity after 24 h	Full-thickness excisional wound	[106]
Chitosan/poly (vinyl pyrrolidone)/poly (N-isopropyl acrylamide)	*Salix alba*	Simple blending	Disk diffusion method	*S. aureus* *E. coli* *P. aeruginosa*	5 mm 4 mm 4 mm	Wound dressing	[107]
Carbopol 980NF/polyethylene glycol	*Rosmarini herba*	Simple blending	Disk diffusion method	*S. aureus* *P. aeruginosa*	10 mm 10 mm	Wound dressing	[96]
Polyvinyl alcohol/pullulan	*Calendula officinalis*	Freeze-thawing	Disk diffusion method	*S. aureus* *E. coli* *P. aeruginosa*	13 ± 0.35 mm 12 ± 0.5 mm 15 ± 0.1 mm	Wound healing	[108]
Cellulose/propylene glycol	*Epilobium angustifolium*	Simple blending	Agar well diffusion method	*S. aureus* *E. coli* *S. epidermidis*	7 ± 0.5 mm 15 ± 0.5 mm 8.5 ± 0.5 mm	-----------------	[109]

## References

[1] Simões D., Miguel S.P., Ribeiro M.P., Coutinho P., Mendonça A.G., Correia I.J. (2018). Recent Advances on Antimicrobial Wound Dressing: A Review. Eur. J. Pharm. Biopharm..

[2] Orchard A., van Vuuren S. (2017). Commercial Essential Oils as Potential Antimicrobials to Treat Skin Diseases. Evid.-Based Complement. Altern. Med..

[3] Orchard A., van Vuuren S.F. (2019). Carrier Oils in Dermatology. Arch. Dermatol. Res..

[4] Mir M., Ali M.N., Barakullah A., Gulzar A., Arshad M., Fatima S., Asad M. (2018). Synthetic Polymeric Biomaterials for Wound Healing: A Review. Prog. Biomater..

[5] Suarato G., Bertorelli R., Athanassiou A. (2018). Borrowing From Nature: Biopolymers and Biocomposites as Smart Wound Care Materials. Front. Bioeng. Biotechnol..

[6] Tchemtchoua V.T., Atanasova G., Aqil A., Filée P., Garbacki N., Vanhooteghem O., Deroanne C., Noël A., Jérome C., Nusgens B. (2011). Development of a Chitosan Nanofibrillar Scaffold for Skin Repair and Regeneration. Biomacromolecules.

[7] Pereira G., Guterres S., Balducci A., Colombo P., Sonvico F. (2014). Polymeric Films Loaded with Vitamin E and *Aloe vera* for Topical Application in the Treatment of Burn Wounds. BioMed Res. Int..

[8] Das U., Behera S.S., Singh S., Rizvi S.I., Singh A.K. (2016). Progress in the Development and Applicability of Potential Medicinal Plant Extract-Conjugated Polymeric Constructs for Wound Healing and Tissue Regeneration. Phyther. Res..

[9] Serov D.A., Khabatova V.V., Vodeneev V., Li R., Gudkov S.V. (2023). A Review of the Antibacterial, Fungicidal and Antiviral Properties of Selenium Nanoparticles. Materials.

[10] Grice E.A., Segre J.A. (2011). The Skin Microbiome. Nat. Rev. Microbiol..

[11] Byrd A.L., Belkaid Y., Segre J.A. (2018). The Human Skin Microbiome. Nat. Rev. Microbiol..

[12] Boxberger M., Cenizo V., Cassir N., La Scola B. (2021). Challenges in Exploring and Manipulating the Human Skin Microbiome. Microbiome.

[13] Hannigan G.D., Grice E.A. (2013). Microbial Ecology of the Skin in the Era of Metagenomics and Molecular Microbiology. Cold Spring Harb. Perspect. Med..

[14] Pereira S.G., Moura J., Carvalho E., Empadinhas N. (2017). Microbiota of Chronic Diabetic Wounds: Ecology, Impact, and Potential for Innovative Treatment Strategies. Front. Microbiol..

[15] Negut I., Grumezescu V., Grumezescu A. (2018). Treatment Strategies for Infected Wounds. Molecules.

[16] Guo S., DiPietro L.A. (2010). Factors Affecting Wound Healing. J. Dent. Res..

[17] Okur M., Karantas I., Ay Z., Üstündağ Okur N., Siafaka P. (2020). Recent Trends on Wound Management: New Therapeutic Choices Based on Polymeric Carriers. Asian J. Pharm. Sci..

[18] Edwards R., Harding K.G. (2004). Bacteria and Wound Healing. Curr. Opin. Infect. Dis..

[19] Cardona A.F., Wilson S.E. (2015). Skin and Soft-Tissue Infections: A Critical Review and the Role of Telavancin in Their Treatment. Clin. Infect. Dis..

[20] Gilliver S.C., Ashworth J.J., Ashcroft G.S. (2007). The Hormonal Regulation of Cutaneous Wound Healing. Clin. Dermatol..

[21] Wilson J., Clark J. (2004). Obesity: Impediment to Postsurgical Wound Healing. Adv. Skin Wound Care.

[22] Parvin F., Vickery K., Deva A.K., Hu H. (2022). Efficacy of Surgical/Wound Washes against Bacteria: Effect of Different In Vitro Models. Materials.

[23] Dhivya S., Padma V.V., Santhini E. (2015). Wound Dressings—A Review. BioMedicine.

[24] Alven S., Khwaza V., Oyedeji O.O., Aderibigbe B.A. (2021). Polymer-Based Scaffolds Loaded with *Aloe vera* Extract for the Treatment of Wounds. Pharmaceutics.

[25] Shah J.B. (2011). The History of Wound Care. J. Am. Col. Certif. Wound Spec..

[26] Daunton C., Kothari S., Smith L.E., Steele D. (2012). A History of Materials and Practices for Wound Management. Wound Pract. Res..

[27] Fu X., Wang Z., Sheng Z. (2001). Advances in Wound Healing Research in China: From Antiquity to the Present. Wound Repair Regen..

[28] Moeini A., Pedram P., Makvandi P., Malinconico M., Gomez d’Ayala G. (2020). Wound Healing and Antimicrobial Effect of Active Secondary Metabolites in Chitosan-Based Wound Dressings: A Review. Carbohydr. Polym..

[29] Koehler J., Brandl F.P., Goepferich A.M. (2018). Hydrogel Wound Dressings for Bioactive Treatment of Acute and Chronic Wounds. Eur. Polym. J..

[30] Taghipour N., Deravi N., Rahimi M. (2020). Chitosan-Based Scaffolds, Suitable Structures for Wound Healing Dressing: A Short Review. J. Regen. Reconstr. Restor. (Triple R).

[31] Micale N., Citarella A., Molonia M.S., Speciale A., Cimino F., Saija A., Cristani M. (2020). Hydrogels for the Delivery of Plant-Derived (Poly)Phenols. Molecules.

[32] Han G., Ceilley R. (2017). Chronic Wound Healing: A Review of Current Management and Treatments. Adv. Ther..

[33] Chattopadhyay S., Raines R.T. (2014). Collagen-Based Biomaterials for Wound Healing. Biopolymers.

[34] Weller C., Team V., Sussman G. (2020). First-Line Interactive Wound Dressing Update: A Comprehensive Review of the Evidence. Front. Pharmacol..

[35] Vyas K., Vasconez H. (2014). Wound Healing: Biologics, Skin Substitutes, Biomembranes and Scaffolds. Healthcare.

[36] Freitas I.R., Cattelan M.G., Holban A.M., Grumezescu A.M. (2018). Chapter 15—Antimicrobial and Antioxidant Properties of Essential Oils in Food Systems—An Overview. Handbook of Food Bioengineering: Volume 10: Microbial Contamination and Food Degradation.

[37] Bashir A., Jabeen S., Gull N., Islam A., Sultan M., Ghaffar A., Khan S., Sagar S., Jamil T. (2018). Co-Concentration Effect of Silane with Natural Extract on Biodegradable Polymeric Films for Food Packaging. Int. J. Biol. Macromol..

[38] Liu T., Liu L., Gong X., Chi F., Ma Z. (2021). Fabrication and Comparison of Active Films from Chitosan Incorporating Different Spice Extracts for Shelf Life Extension of Refrigerated Pork. LWT.

[39] Mombeshora M., Mukanganyama S. (2019). Antibacterial Activities, Proposed Mode of Action and Cytotoxicity of Leaf Extracts from *Triumfetta welwitschii* against *Pseudomonas aeruginosa*. BMC Complement. Altern. Med..

[40] Criollo-Mendoza M.S., Contreras-Angulo L.A., Leyva-López N., Gutiérrez-Grijalva E.P., Jiménez-Ortega L.A., Heredia J.B. (2023). Wound Healing Properties of Natural Products: Mechanisms of Action. Molecules.

[41] Zuzarte M., Gonçalves M., Canhoto J., Salgueiro L., A. Méndez-Vilas (2011). Antidermatophytic Activity of Essential Oils. Science against Microbial Pathogens: Communicating Current Research and Technological Advances.

[42] Acosta S., Chiralt A., Santamarina Siurana M., Roselló J., Gonzalez-Martinez C., Cháfer M. (2016). Antifungal Films Based on Starch-Gelatin Blend, Containing Essential Oils. Food Hydrocoll..

[43] Burt S., Van der Zee R., Koets A., Graaff A., Knapen F., Gaastra W., Haagsman H., Veldhuizen E. (2007). Carvacrol Induces Heat Shock Protein 60 and Inhibits Synthesis of Flagellin in *Escherichia coli* O157:H7. Appl. Environ. Microbiol..

[44] Nazzaro F., Fratianni F., De Martino L., Coppola R., De Feo V. (2013). Effect of Essential Oils on Pathogenic Bacteria. Pharmaceuticals.

[45] Burt S. (2004). Essential Oils: Their Antibacterial Properties and Potential Applications in Foods—A Review. Int. J. Food Microbiol..

[46] Nazzaro F., Fratianni F., Coppola R. (2013). Quorum Sensing and Phytochemicals. Int. J. Mol. Sci..

[47] Sadiq M.B., Tarning J., Cho T.Z.A., Anal A.K. (2017). Activities and Possible Modes of Action of *Acacia nilotica* (L.) Del. against Multidrug-Resistant *Escherichia coli* and *Salmonella*. Molecules.

[48] Chew Y.L., Mahadi A.M., Wong K.M., Goh J.K. (2018). Anti-Methicillin-Resistance *Staphylococcus aureus* (MRSA) Compounds from *Bauhinia kockiana* Korth. and Their Mechanism of Antibacterial Activity. BMC Complement. Altern. Med..

[49] Álvarez-Martínez F.J., Rodríguez J.C., Borrás-Rocher F., Barrajón-Catalán E., Micol V. (2021). The Antimicrobial Capacity of *Cistus salviifolius* and *Punica granatum* Plant Extracts against Clinical Pathogens Is Related to Their Polyphenolic Composition. Sci. Rep..

[50] Zhou D., Liu Z.-H., Wang D.-M., Li D.-W., Yang L.-N., Wang W. (2019). Chemical Composition, Antibacterial Activity and Related Mechanism of Valonia and Shell from *Quercus variabilis* Blume (Fagaceae) against *Salmonella paratyphi* a and *Staphylococcus aureus*. BMC Complement. Altern. Med..

[51] Xu M., Xue H., Li X., Zhao Y., Lin L., Yang L., Zheng G. (2019). Chemical Composition, Antibacterial Properties, and Mechanism of *Smilax china* L. Polyphenols. Appl. Microbiol. Biotechnol..

[52] Roshan N., Riley T.V., Knight D.R., Steer J.H., Hammer K.A. (2019). Natural Products Show Diverse Mechanisms of Action against *Clostridium difficile*. J. Appl. Microbiol..

[53] Maisetta G., Batoni G., Caboni P., Esin S., Rinaldi A.C., Zucca P. (2019). Tannin Profile, Antioxidant Properties, and Antimicrobial Activity of Extracts from Two Mediterranean Species of Parasitic Plant *Cytinus*. BMC Complement. Altern. Med..

[54] Kozics K., Bučková M., Puškárová A., Kalászová V., Cabicarová T., Pangallo D. (2019). The Effect of Ten Essential Oils on Several Cutaneous Drug-Resistant Microorganisms and Their Cyto/Genotoxic and Antioxidant Properties. Molecules.

[55] Chouhan S., Sharma K., Guleria S. (2017). Antimicrobial Activity of Some Essential Oils—Present Status and Future Perspectives. Medicines.

[56] Owen L., White A.W., Laird K. (2019). Characterisation and Screening of Antimicrobial Essential Oil Components against Clinically Important Antibiotic-resistant Bacteria Using Thin Layer Chromatography-direct Bioautography Hyphenated with GC-MS, LC-MS and NMR. Phytochem. Anal..

[57] Ivanovic J., Misic D., Zizovic I., Ristic M. (2012). In Vitro Control of Multiplication of Some Food-Associated Bacteria by Thyme, Rosemary and Sage Isolates. Food Control.

[58] Nayim P., Mbaveng A.T., Wamba B.E.N., Fankam A.G., Dzotam J.K., Kuete V. (2018). Antibacterial and Antibiotic-Potentiating Activities of Thirteen Cameroonian Edible Plants against Gram-Negative Resistant Phenotypes. Sci. World J..

[59] Fernandes A., Rodrigues P.M., Pintado M., Tavaria F.K. (2023). A Systematic Review of Natural Products for Skin Applications: Targeting Inflammation, Wound Healing, and Photo-Aging. Phytomedicine.

[60] Yang K., Han Q., Chen B., Zheng Y., Zhang K., Li Q., Wang J. (2018). Antimicrobial Hydrogels: Promising Materials for Medical Application. Int. J. Nanomed..

[61] Naseri-Nosar M., Ziora Z.M. (2018). Wound Dressings from Naturally-Occurring Polymers: A Review on Homopolysaccharide-Based Composites. Carbohydr. Polym..

[62] Rani Raju N., Silina E., Stupin V., Manturova N., Chidambaram S.B., Achar R.R. (2022). Multifunctional and Smart Wound Dressings—A Review on Recent Research Advancements in Skin Regenerative Medicine. Pharmaceutics.

[63] Savencu I., Iurian S., Porfire A., Bogdan C., Tomuță I. (2021). Review of Advances in Polymeric Wound Dressing Films. React. Funct. Polym..

[64] Avila-Sosa R., Palou E., López-Malo A., Preedy V.R. (2016). Chapter 15—Essential Oils Added to Edible Films. Essential Oils in Food Preservation, Flavor and Safety.

[65] Borbolla-Jiménez F.V., Peña-Corona S.I., Farah S.J., Jiménez-Valdés M.T., Pineda-Pérez E., Romero-Montero A., Del Prado-Audelo M.L., Bernal-Chávez S.A., Magaña J.J., Leyva-Gómez G. (2023). Films for Wound Healing Fabricated Using a Solvent Casting Technique. Pharmaceutics.

[66] Pérez-Recalde M., Arias I.E.R., Hermida É.B. (2018). Could Essential Oils Enhance Biopolymers Performance for Wound Healing? A Systematic Review. Phytomedicine.

[67] Ellis S., Lin E.J., Tartar D. (2018). Immunology of Wound Healing. Curr. Dermatol. Rep..

[68] Atarés L., Chiralt A. (2016). Essential Oils as Additives in Biodegradable Films and Coatings for Active Food Packaging. Trends Food Sci. Technol..

[69] Augusto A., Dias J.R., Campos M.J., Alves N.M., Pedrosa R., Silva S.F.J. (2018). Influence of *Codium tomentosum* Extract in the Properties of Alginate and Chitosan Edible Films. Foods.

[70] Antunes J.C., Tavares T.D., Teixeira M.A., Teixeira M.O., Homem N.C., Amorim M.T.P., Felgueiras H.P. (2021). Eugenol-Containing Essential Oils Loaded onto Chitosan/Polyvinyl Alcohol Blended Films and Their Ability to Eradicate *Staphylococcus aureus* or *Pseudomonas aeruginosa* from Infected Microenvironments. Pharmaceutics.

[71] Güneş S., Tıhmınlıoğlu F. (2017). Hypericum Perforatum Incorporated Chitosan Films as Potential Bioactive Wound Dressing Material. Int. J. Biol. Macromol..

[72] Liakos I., Rizzello L., Scurr D., Pompa P., Athanassiou A. (2014). All-Natural Composite Wound Dressing Films of Essential Oils Encapsulated in Sodium Alginate with Antimicrobial Properties. Int. J. Pharm..

[73] Hafsa J., ali Smach M., Ben Khedher M.R., Charfeddine B., Limem K., Majdoub H., Rouatbi S. (2016). Physical, Antioxidant and Antimicrobial Properties of Chitosan Films Containing *Eucalyptus globulus* Essential Oil. LWT Food Sci. Technol..

[74] Wang L., Liu F., Jiang Y., Chai Z., Li P., Cheng Y., Jing H., Leng X. (2011). Synergistic Antimicrobial Activities of Natural Essential Oils with Chitosan Films. J. Agric. Food Chem..

[75] Ge Y., Ge M. (2015). Sustained Broad-Spectrum Antimicrobial and Haemostatic Chitosan-Based Film with Immerged Tea Tree Oil Droplets. Fibers Polym..

[76] Altiok D., Altiok E., Tihminlioglu F. (2010). Physical, Antibacterial and Antioxidant Properties of Chitosan Films Incorporated with Thyme Oil for Potential Wound Healing Applications. J. Mater. Sci. Mater. Med..

[77] Dos Santos E.P., Nicácio P.H.M., Barbosa F.C., da Silva H.N., Andrade A.L.S., Fook M.V.L., de Silva S.M., Lima I.F.L. (2019). Chitosan/Essential Oils Formulations for Potential Use as Wound Dressing: Physical and Antimicrobial Properties. Materials.

[78] Râpă M., Zaharescu T., Stefan L.M., Gaidău C., Stănculescu I., Constantinescu R.R., Stanca M. (2022). Bioactivity and Thermal Stability of Collagen–Chitosan Containing Lemongrass Essential Oil for Potential Medical Applications. Polymers.

[79] Valencia-Gómez L.E., Martel-Estrada S.A., Vargas-Requena C., Rivera-Armenta J.L., Alba-Baena N., Rodríguez-González C., Olivas-Armendáriz I. (2016). Chitosan/*Mimosa tenuiflora* Films as Potential Cellular Patch for Skin Regeneration. Int. J. Biol. Macromol..

[80] Valencia-Gómez L., Martel-Estrada S.-A., Vargas-Requena C.-L., Acevedo-Fernández J.-J., Rodriguez C., Hernández-Paz J.-F., Santos-Rodríguez E., Olivas-Armendariz I. (2019). Characterization and Evaluation of a Novel O-Carboxymethyl Chitosan Films with *Mimosa tenuiflora* Extract for Skin Regeneration and Wound Healing. J. Bioact. Compat. Polym..

[81] Ghafoor B., Ali M.N., Ansari U., Bhatti M., Akhtar H., Mir M., Darkhsan F. (2016). New Biofunctional Loading of Natural Antimicrobial Agent in Biodegradable Polymeric Films for Biomedical Applications. Int. J. Biomater..

[82] Costa N.N., de Faria Lopes L., Ferreira D.F., de Prado E.M.L., Severi J.A., Resende J.A., de Paula Careta F., Ferreira M.C.P., Carreira L.G., de Souza S.O.L. (2020). Polymeric Films Containing Pomegranate Peel Extract Based on PVA/Starch/PAA Blends for Use as Wound Dressing: In Vitro Analysis and Physicochemical Evaluation. Mater. Sci. Eng. C.

[83] Muthukumar T., Senthil R., Sastry T.P. (2013). Synthesis and Characterization of Biosheet Impregnated with *Macrotyloma uniflorum* Extract for Burn/Wound Dressings. Colloids Surf. B Biointerfaces.

[84] Agarwal R., Alam M.S., Gupta B. (2013). Preparation of Curcumin Loaded Poly(vinyl alcohol)-Poly(ethylene oxide)-carboxymethyl Cellulose Membranes for Wound Care Application. J. Biomater. Tissue Eng..

[85] Mutlu B., Erci F., Çakır Koç R. (2022). Production of Alginate Films Containing *Hypericum perforatum* Extract as an Antibacterial and Antioxidant Wound Dressing Material. J. Bioact. Compat. Polym..

[86] Cruz Sánchez E., García M.T., Pereira J., Oliveira F., Craveiro R., Paiva A., Gracia I., García-Vargas J.M., Duarte A.R.C. (2023). Alginate–Chitosan Membranes for the Encapsulation of Lavender Essential Oil and Development of Biomedical Applications Related to Wound Healing. Molecules.

[87] Sathuvan M., Thangam R., Cheong K.-L., Kang H., Liu Y. (2023). κ-Carrageenan-Essential Oil Loaded Composite Biomaterial Film Facilitates Mechanosensing and Tissue Regenerative Wound Healing. Int. J. Biol. Macromol..

[88] Harrison I., Spada F. (2018). Hydrogels for Atopic Dermatitis and Wound Management: A Superior Drug Delivery Vehicle. Pharmaceutics.

[89] Weller C., Weller C., Team V., Rajendran S. (2019). 4—Interactive Dressings and Their Role in Moist Wound Management. Advanced Textiles for Wound Care.

[90] Lee S.C., Kwon I.K., Park K. (2013). Hydrogels for Delivery of Bioactive Agents: A Historical Perspective. Adv. Drug Deliv. Rev..

[91] Wichterle O., Lím D. (1960). Hydrophilic Gels for Biological Use. Nature.

[92] Buwalda S.J., Boere K.W.M., Dijkstra P.J., Feijen J., Vermonden T., Hennink W.E. (2014). Hydrogels in a Historical Perspective: From Simple Networks to Smart Materials. J. Control. Release.

[93] Chummun I., Bekah D., Goonoo N., Bhaw-Luximon A. (2021). Assessing the Mechanisms of Action of Natural Molecules/Extracts for Phase-Directed Wound Healing in Hydrogel Scaffolds. RSC Med. Chem..

[94] Zagórska-Dziok M., Sobczak M. (2020). Hydrogel-Based Active Substance Release Systems for Cosmetology and Dermatology Application: A Review. Pharmaceutics.

[95] Mahmood H., Ullah Khan I., Asif M., Khan R., Asghar S., Khalid I., Syed H., Irfan M., Rehman F., Shahzad Y. (2021). In Vitro and In Vivo Evaluation of Gellan Gum Hydrogel Films: Assessing the Co Impact of Therapeutic Oils and Ofloxacin on Wound Healing. Int. J. Biol. Macromol..

[96] Gavan A., Colobatiu L., Hanganu D., Bogdan C., Olah N.-K., Achim M., Simona M. (2022). Development and Evaluation of Hydrogel Wound Dressings Loaded with Herbal Extracts. Processes.

[97] Wang H., Liu Y., Cai K., Zhang B., Tang S., Zhang W., Liu W. (2021). Antibacterial Polysaccharide-Based Hydrogel Dressing Containing Plant Essential Oil for Burn Wound Healing. Burns Trauma.

[98] Cai K., Liu Y., Yue Y., Liu Y., Guo F. (2023). Essential Oil Nanoemulsion Hydrogel with Anti-Biofilm Activity for the Treatment of Infected Wounds. Polymers.

[99] Chuysinuan P., Chimnoi N., Reuk-Ngam N., Khlaychan P., Makarasen A., Wetprasit N., Dechtrirat D., Supaphol P., Techasakul S. (2019). Development of Gelatin Hydrogel Pads Incorporated with *Eupatorium adenophorum* Essential Oil as Antibacterial Wound Dressing. Polym. Bull..

[100] Altaf F., Niazi M.B.K., Jahan Z., Ahmad T., Akram M.A., Safdar A., Butt M.S., Noor T., Sher F. (2021). Synthesis and Characterization of PVA/Starch Hydrogel Membranes Incorporating Essential Oils Aimed to Be Used in Wound Dressing Applications. J. Polym. Environ..

[101] Singh P., Verma C., Mukhopadhyay S., Gupta A., Gupta B. (2022). Preparation of Thyme Oil Loaded κ-Carrageenan-Polyethylene Glycol Hydrogel Membranes as Wound Care System. Int. J. Pharm..

[102] Kavoosi G., Bordbar Z., Dadfar S.M., Dadfar S.M.M. (2017). Preparation and Characterization of a Novel Gelatin–Poly(vinyl alcohol) Hydrogel Film Loaded with *Zataria multiflora* Essential Oil for Antibacterial–Antioxidant Wound-Dressing Applications. J. Appl. Polym. Sci..

[103] Sami D.G., Abdellatif A., Azzazy H.M.E. (2020). Turmeric/Oregano Formulations for Treatment of Diabetic Ulcer Wounds. Drug Dev. Ind. Pharm..

[104] Annapoorna M., Kumar P.T., Lakshman L.R., Lakshmanan V.-K., Nair S.V., Jayakumar R. (2013). Biochemical Properties of Hemigraphis Alternata Incorporated Chitosan Hydrogel Scaffold. Carbohydr. Polym..

[105] Chanaj-Kaczmarek J., Paczkowska M., Osmałek T., Kaprón B., Plech T., Szymanowska D., Karazniewicz-Łada M., Kobus-Cisowska J., Cielecka-Piontek J. (2020). Hydrogel Delivery System Containing *Calendulae flos* Lyophilized Extract with Chitosan as a Supporting Strategy for Wound Healing Applications. Pharmaceutics.

[106] Salehi M., Zamiri S., Samadian H., Ai J., Foroutani L., Ai A., Khanmohammadi M. (2021). Chitosan Hydrogel Loaded with *Aloe vera* Gel and Tetrasodium Ethylenediaminetetraacetic Acid (EDTA) as the Wound Healing Material: In Vitro and in Vivo Study. J. Appl. Polym. Sci..

[107] Qureshi M.A., Khatoon F., Rizvi M.A., Zafaryab M. (2015). Ethyl Acetate *Salix alba* Leaves Extract-Loaded Chitosan-Based Hydrogel Film for Wound Dressing Applications. J. Biomater. Sci. Polym. Ed..

[108] Pelin I.M., Silion M., Popescu I., Rîmbu C.M., Fundueanu G., Constantin M. (2023). Pullulan/Poly(vinyl alcohol) Hydrogels Loaded with *Calendula officinalis* Extract: Design and In Vitro Evaluation for Wound Healing Applications. Pharmaceutics.

[109] Nowak A., Zagórska-Dziok M., Perużyńska M., Cybulska K., Kucharska E., Ossowicz-Rupniewska P., Piotrowska K., Duchnik W., Kucharski Ł., Sulikowski T. (2022). Assessment of the Anti-Inflammatory, Antibacterial and Anti-Aging Properties and Possible Use on the Skin of Hydrogels Containing *Epilobium angustifolium* L. Extracts. Front. Pharmacol..

[110] Rathod L., Bhowmick S., Patel P., Sawant K. (2022). Calendula Flower Extract Loaded PVA Hydrogel Sheet for Wound Management: Optimization, Characterization and In-Vivo Study. J. Drug Deliv. Sci. Technol..

[111] Alsakhawy S.A., Baghdadi H.H., El-Shenawy M.A., Sabra S.A., El-Hosseiny L.S. (2022). Encapsulation of *Thymus vulgaris* Essential Oil in Caseinate/Gelatin Nanocomposite Hydrogel: In Vitro Antibacterial Activity and In Vivo Wound Healing Potential. Int. J. Pharm..

[112] Ali A., Garg P., Goyal R., Khan A., Negi P., Li X., Kulshrestha S. (2022). An Efficient Wound Healing Hydrogel Based on a Hydroalcoholic Extract of MORINGA OLEIFERA Seeds. S. Afr. J. Bot..

[113] Casado-Diaz A., Moreno-Rojas J.M., Verdú-Soriano J., Lázaro-Martínez J.L., Rodríguez-Mañas L., Tunez I., La Torre M., Berenguer Pérez M., Priego-Capote F., Pereira-Caro G. (2022). Evaluation of Antioxidant and Wound-Healing Properties of EHO-85, a Novel Multifunctional Amorphous Hydrogel Containing *Olea europaea* Leaf Extract. Pharmaceutics.

[114] Khan B.A., Khan A., Khan M.K., Braga V.A. (2021). Preparation and Properties of High Sheared Poly(vinyl alcohol)/Chitosan Blended Hydrogels Films with *Lawsonia inermis* Extract as Wound Dressing. J. Drug Deliv. Sci. Technol..

[115] Antonescu I.A., Antonescu A., Miere F., Fritea L., Teușdea A.C., Vicaș L., Vicaș S.I., Brihan I., Domuța M., Zdrinca M. (2021). Evaluation of Wound Healing Potential of Novel Hydrogel Based on *Ocimum basilicum* and *Trifolium pratense* Extracts. Processes.

